# Reduced NOV expression correlates with disease progression in colorectal cancer and is associated with survival, invasion and chemoresistance of cancer cells

**DOI:** 10.18632/oncotarget.15439

**Published:** 2017-02-17

**Authors:** Jun Li, Lin Ye, Ping-Hui Sun, Fei Zheng, Fiona Ruge, Lucy K. Satherley, Yi Feng, Huishan Zhao, Guifang Du, Tingting Wang, Yao Yang, Xuemei Ma, Shan Cheng, Xiaomei Yang, Hefen Yu, Xu Teng, Yang Si, Zhongtao Zhang, Wen G. Jiang

**Affiliations:** ^1^ Department of General Surgery, Beijing Key Laboratory of Cancer Invasion and Metastasis Research and National Clinical Research Center for Digestive Diseases, Beijing Friendship Hospital, Capital Medical University, Beijing 100050, China; ^2^ Cardiff China Medical Research Collaborative, Cardiff University School of Medicine, Heath Park, Cardiff, CF14 4XN, UK; ^3^ Cardiff University, Capital Medical University Joint Centre for Biomedical Research and Cancer Institute, Capital Medical University, Beijing, 100069, China

**Keywords:** NOV, colorectal cancer, JNK signalling pathways, proliferation, invasion

## Abstract

Aberrant expression of nephroblastoma overexpressed (NOV) has been evident in certain malignancies. In the current study, we aim to investigate the role played by NOV in colorectal cancer (CRC). NOV expression was determined in a cohort of 359 CRC tissues and 174 normal colorectal tissues. Its impact on CRC cells was investigated using *in vitro* NOV knockdown and overexpression models. NOV transcripts were reduced in the CRC tumours compared with the paired adjacent normal colorectal tissues (*p* < 0.01) and was associated with distant metastases. NOV knockdown resulted in increased cell proliferation and invasion of RKO cells, whilst an opposite effect was seen in the HT115 NOV over expressing cells. A positive association between Caspase-3/-8 and NOV was seen in NOV knockdown and overexpression cell lines which contributed to the survival of serum deprived CRC cells. Further investigation showed that NOV regulated proliferation, survival and invasion through the JNK pathway. NOV knockdown in RKO cells reduced the responsiveness to 5-Fluorouracil treatment, whilst overexpression in HT115 cells exhibited a contrasting effect. Taken together, NOV is reduced in CRC tumours and this is associated with disease progression. NOV inhibits the proliferation and invasion of CRC cells *in vitro*. Inhibition of proliferation is mediated by a regulation of Caspase-3/-8, via the JNK pathway, which has potential for predicting and preventing chemoresistance.

## INTRODUCTION

Worldwide, colorectal cancer (CRC) is the second most commonly diagnosed cancer in females and third most commonly diagnosed cancer in males. An estimated 1.2 million new cases, and 608,700 deaths due to CRC occurred in 2008 [1]. The highest incidence rates have been documented in Australia and New Zealand, Europe, and North America, whereas the lowest rates have been reported in Africa and South-Central Asia [1]. In China, the National Central Cancer Registry (NCCR) information indicated that colorectal cancer is the fourth most common cause of cancer death in women and fifth most common cause of cancer death in males [2].

The CCN family derives its name from an acronym of cysteine-rich protein 61 (CYR61), connective tissue growth factor (CTGF) and nephroblastoma overexpressed (NOV), which were the first three members discovered in the early 1990's [2–4]. These family members have since been renamed CCN1, 2 and 3 respectively, whilst the subsequently discovered other three family members; Wnt-induced secreted protein-1 (WISP-1), WISP-2, and WISP-3 are also now known as CCN4-6 respectively [5]. The CCNs are a group of matricellular signalling molecules, similar to other extracellular matrix proteins, involved in embryonic and postnatal development, angiogenesis, wound healing, fibrosis and inflammation [6–10].

An emerging role has been revealed for CCNs in a variety of malignancies. An elevated expression of CCN1 protein has been indicated in invasive ductal carcinomas compared to ductal and lobular carcinoma *in situ* [11]. Our previous study revealed strong immunohistochemical staining of CCN4, CCN5 and CCN6 in normal colorectal epithelial cells, which was confined mostly to the cell membrane with a weaker staining present in the stroma. Membrane staining of CCN4, CCN5 and CCN6 were reduced in CRC tumours, with an elevated cytoplasmic staining of CCN4 and CCN6 but not CCN5 [12].

The NOV gene codes a protein (CCN3) of 357 amino acids with an N-terminal secretory signal peptide and four functional domains: insulin-like growth factor binding protein (IGFBP), von Willebrand factor C (VWC), thrombospondin 1 (TSP-1) and a C-terminal cysteine knot (CT) [13]. Similar to other CCN members, overexpression of NOV has been observed in a number of solid tumours. Increased expression of NOV has been seen in prostate cancer cell lines compared with immortalized prostatic epithelial cell lines [14]. Primary musculoskeletal tumours that developed lung and/or bone metastases have been found to express a higher level of NOV [15]. NOV transcripts and protein levels have also been observed to be increased in cervical cancer tissues compared with corresponding normal tissues. The overexpression of CCN3 in cervical cancer was significantly associated with disease progression and lymph node metastasis [16]. A recent study reported elevated expression of NOV in a cohort of 126 CRC specimens [17]. However, the role played by NOV in colorectal cancer (CRC) remains unclear. The current study aims to investigate the role played by NOV in CRC.

## RESULTS

### The expression of NOV is reduced in CRC

We first examined the expression of NOV in a cohort of CRC tissues, which included 359 CRC tumours and 174 paired adjacent normal colorectal tissues, using real time PCR (Table 1). Reduced levels of NOV transcripts were seen in CRC tumours compared with its expression in the adjacent normal colorectal tissues (*p* = 0.0024). In analyses of two public available gene expression array data of human CRC tissue samples, reduced expression of NOV was also seen CRC tumours in comparison with normal colon tissue (Supplementary Figure 1A) or paired adjacent normal colon tissues (Supplementary Figure 1B). Reduced levels of NOV transcripts were seen in patients with distant metastases compared with that of patients who remained disease free (*p* = 0.012). The NOV transcript levels were found to be lower in rectal tumours in comparison with that seen in colon tumours (*p* = 0.0046). However, NOV transcripts were higher in tumours with more invasive growth/expansion which had invaded through the muscularis propria including T3 and T4 tumours, according to the TNM staging, in comparison to the expression in T1 and T2 tumours (*p* < 0.01). There were no correlations observed between NOV expression, tumour differentiation and lymphatic metastases.

**Table 1:** NOV transcript levels in CRC

Category		No.	Means ± SE (copies)	*P*-value
T/N	Normal	174	22008 ± 6994	
	Tumour	359	437 ± 126	**0.0024**
Paired T-N	Paired T-N	173	T-N:-21295 ± 7039	**0.0029**
Location	Colon	212	689 ± 2108	
	Rectum	144	72.4 ± 48.10	**0.0046**
Differentiation	Well-differentiated	71	669 ± 358	
	Moderately differentiated	207	131.8 ± 42.1	0.35
	Poorly differentiated	35	1480 ± 780	0.094
Tumour stage	T1	1	*	
	T2	27	53 ± 23.2	
	T3	111	558 ± 256	T2 vs T3: 0.052
	T4	193	327 ± 136	**T2 vs T4: 0.049**
	T1&2	28	51 ± 22.5	
	T3&4	304	304 ± 127	**T1 & 2 vs T3 & 4: 0.0056**
Lymph node	N0	169	354 ± 153	
	N1	94	435 ± 233	0.77
	N2	69	374 ± 273	0.95
	N1&2	163	409 ± 177	0.81
Metastasis	M0	301	413 ± 128	
	M1	31	74 ± 37.1	**0.012**
TNM stage	I	21	68 ± 29.2	
	II	144	405 ± 179	0.066
	III	136	474 ± 212	0.060
	IV	31	74 ± 37.1	0.91
	II & III & IV	311	402 ± 124	**0.0093**

**Note:** There were some missing cases due to lack of record of relevant information, including 3 cases missed for location, 46 cases for differentiation and 27 cases for TNM staging. For TNM staging, TNM III and IV groups had 167 cases including 4 N0 cases which had distant metastases, while the rest were N1 or N2.

**Figure 1:**
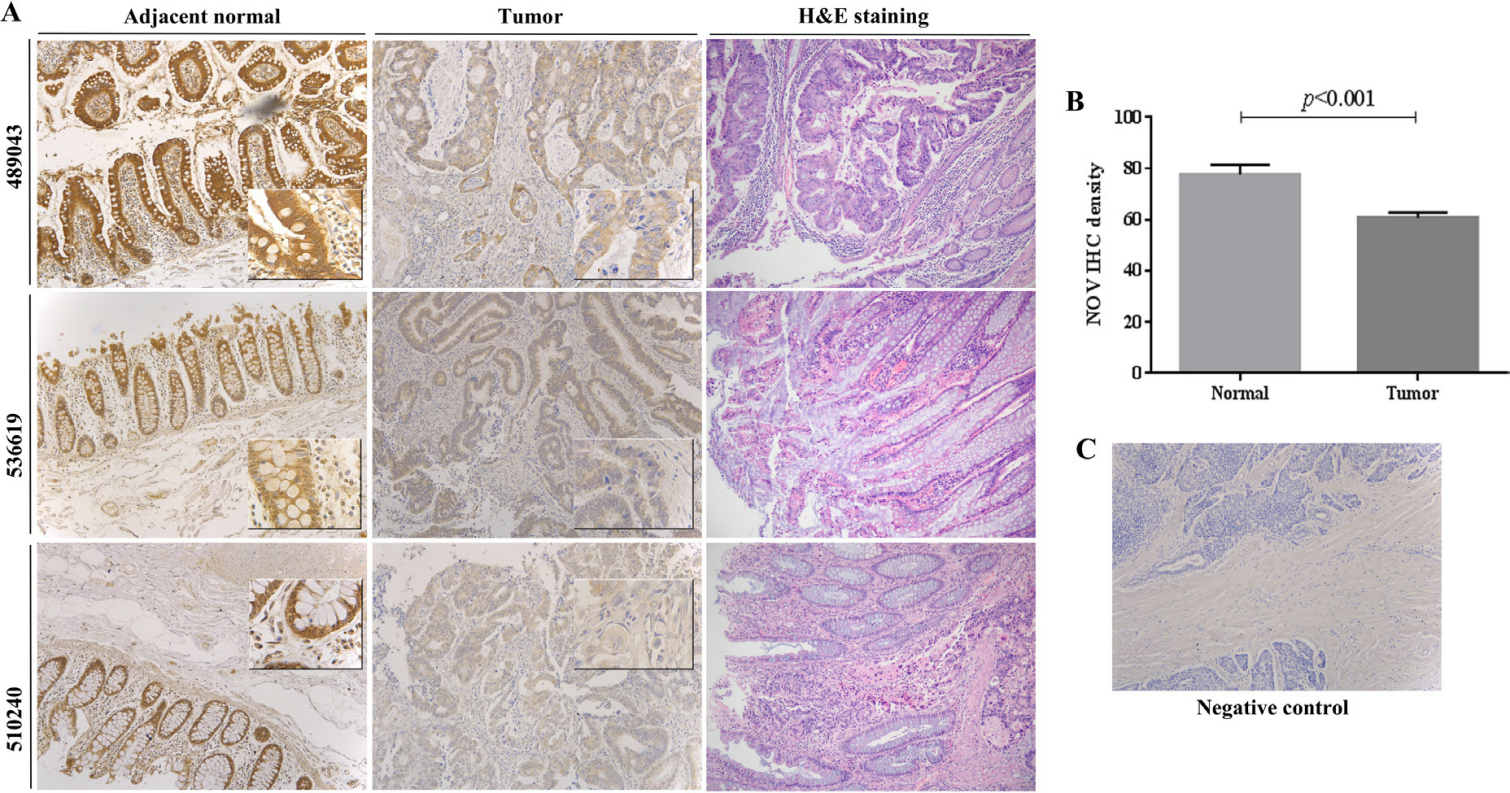
Expression of NOV protein in CRC (**A**) The expression of NOV protein in human CRC tumours were assessed using immunochemical staining in comparison with paired adjacent normal colon tissues. H&E staining of the same sample is also provided. The images were reduced from × 100 magnification. Small panels, inserted in main images, were reduced from a photo taken under × 400 magnification. (**B**) Semi-quantification of NOV staining in CRC tissues. Shown are average intensity of NOV staining in the tumours compared with its staining in the adjacent normal colorectal tissues. Error bars represent standard deviation. (**C**) Negative control, where samples were treated with secondary antibody only is included.

We further determined the expression of NOV protein in CRC tumours through immunochemical staining of NOV in 45 CRC tumours and 27 adjacent normal colorectal tissues (Figure 1). Intensive staining of NOV was observed in the cytoplasm of epithelial cells in normal colon tissues and the staining was reduced in CRC tissues. No differential expression of NOV was seen in the tumours of different T stages and also those that had either lymph node metastases or distant metastases (Supplementary Figure 2). Additional information is provided in Supplementary Table 1.

### Impact of altered NOV expression on the adhesion, migration and invasion of CRC cells

**Figure 2:**
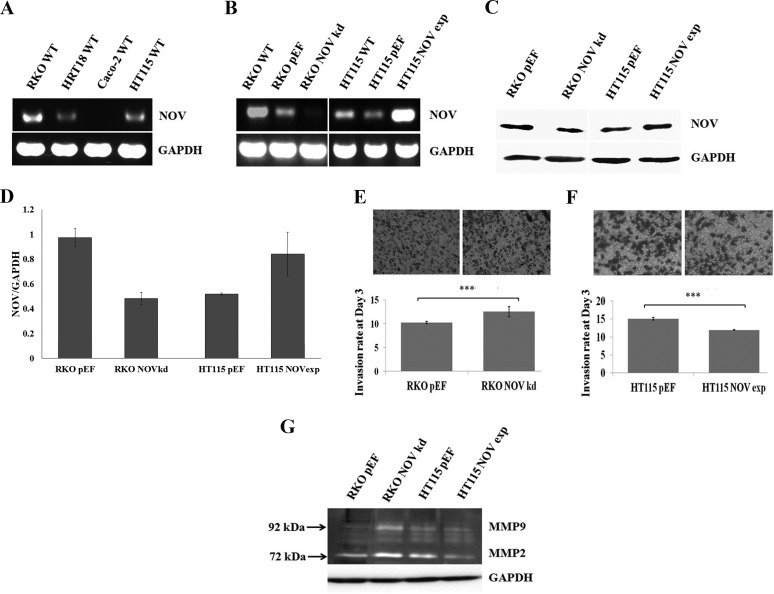
NOV expression in CRC cell lines and cancer cell invasion (**A**) NOV expression in the CRC cell lines was examined using RT-PCR. (**B**) Knockdown and overexpression of NOV in CRC cell lines were verified using RT-PCR. (**C**) Corresponding changes of the NOV protein in the transfected cell lines were further confirmed using Western blot analysis. (**D**) Three independent transfections were performed for each cell line. Shown are NOV/GAPDH ratio using semi-quantitative analysis of the Western blot data. Error bars are standard error of mean. (**E**) An increased invasion was seen in the RKO^NOVkd^ cells compared with RKO^pEF^ cells (*p* < 0.001). (**F**) NOV overexpression reduced the invasiveness of HT115 cells (*p* < 0.001). (**G**) The activation of MMPs were determined using a gelatine zymography. *** represents *p* < 0.001. Three independent experiments were performed. Shown are representative results, error bars represent standard deviations.

To establish an *in vitro* cell line model for exploring the implications of NOV in CRC, we first examined the expression of NOV in a panel of CRC cell lines, i.e. RKO, HRT18, Caco-2 and HT115 using conventional PCR (Figure 2A). NOV was highly expressed by RKO cells compared with HRT18 and HT115 cell lines and it was absent from Caco2 cells. For assessing the effect of NOV on cellular functions, knockdown of NOV was performed in the RKO cells, while HT115 cells were used to generate a NOV overexpression model. Knockdown and overexpression of NOV in transfected cells was verified using RT-PCR (Figure 2B) and Western blotting (Figure 2C and 2D).

Following verification, we examined the influence on cell-matrix adhesion. Neither knockdown nor overexpression of NOV altered the adhesion of CRC cells compared with the controls (Supplementary Figure 3A). A wound healing assay was also employed to examine the impact of NOV on the migration of the CRC cell lines. No obvious change was observed (Supplementary Figure 3B). We then determined the invasiveness of those transfected cells: knockdown of NOV in RKO cells (RKO^NOVkd^) resulted in an increased invasion compared with the control cells (RKO^pEF^) (*p* < 0.001) (Figure 2E), whilst the HT115^NOVexp^ exhibited reduced cell invasiveness (*p* < 0.001) (Figure 2F). NOV has been reported to be able to repress the transcription and activation of matrix metalloproteinases (MMPs), thus inhibiting the invasion of melanoma cells [18]. In order to investigate the activity of MMPs in these CRC cells, we used gelatin zymography to analyse their activity. Knockdown of NOV resulted in an increase of both active MMP2 and MMP9 in RKO cells, which was consistent with the enhanced invasiveness, while a reduced activation of MMP2 was seen in the HT115^NOVexp^ cells (Figure 2G).

### Effect of NOV knockdown and overexpression on cell proliferation and survival

**Figure 3:**
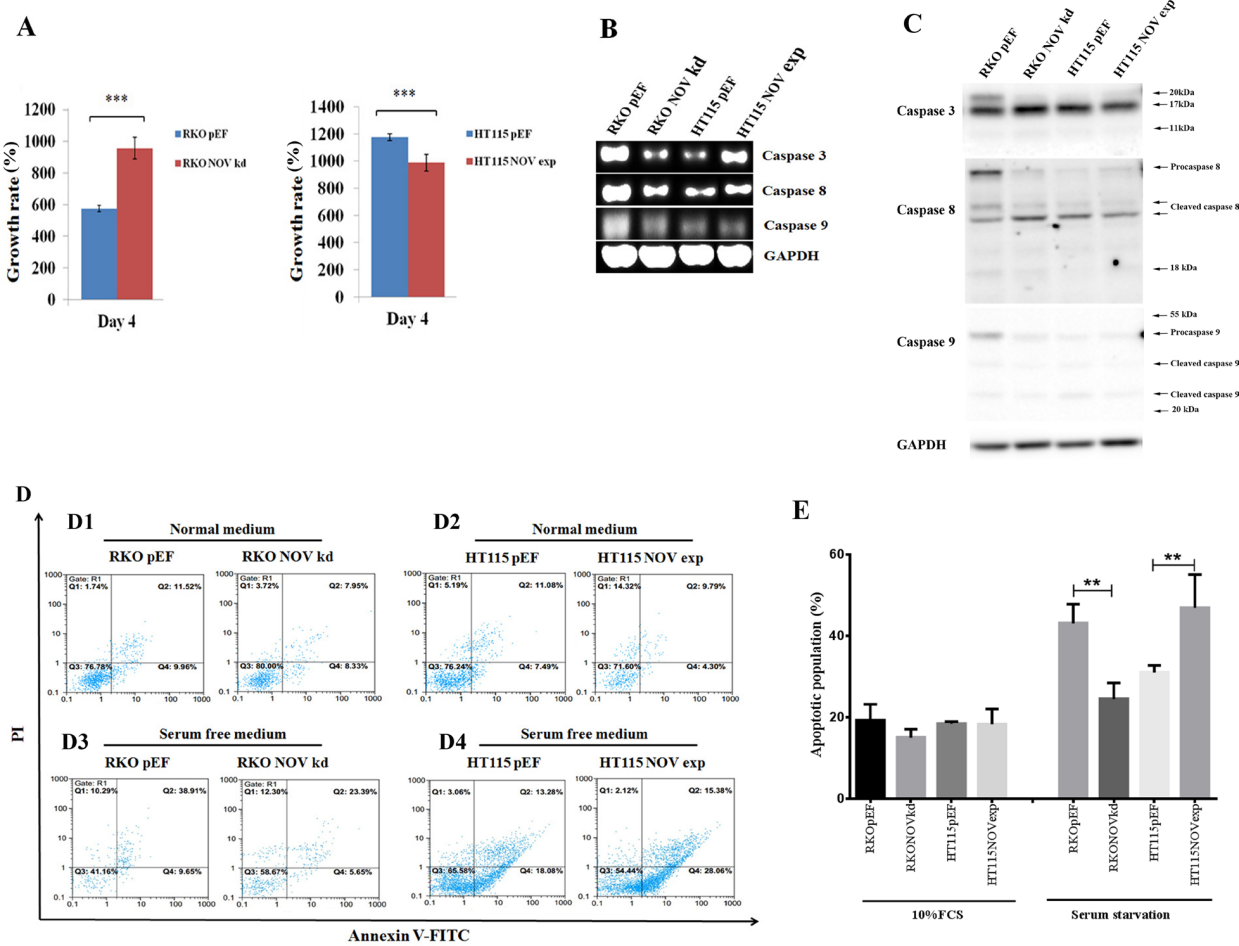
NOV suppresses proliferation and survival of CRC cell lines (**A**) Proliferation of CRC cell lines with altered expression of NOV was determined using an *in vitro* proliferation assay. The proliferation over a period of up to four days was assessed for RKO^NOVkd^ cells and HT115^NOVexp^ cells in comparison with their corresponding control. Three independent experiments were performed. Shown are representative results, error bars represent standard deviations. *** represents *p* < 0.001, ** represents *p* < 0.01. (**B**) Expression of Caspase -3, -8 and -9 in the RKO and HT115 cells were examined using RT-PCR. (**C**) Western blots were employed to detect protein expression and cleavage of caspases-3, -8 and -9. Pro-caspase 3 is approximately 32kDa and is cleaved into 17kDa and 12 kDa subunits. Pro-caspase 8 is approximately 57kDa and can be cleaved into 43kDa, 41kDa and 18kDa subunits. Pro-caspase 9 was detected as bands around 45kDa, and subunits at sizes of approximately 35kDa and 25kDa following cleavage. (**D**) A flow cytometric apoptosis assay was employed to analyze the apoptotic populations in the CRC cell lines under normal culture condition using DMEM supplemented with 10% foetal bovine serum (C1:RKO and C2:HT115) or deprived from the serum (C3:RKO and C4:HT115). Q3 were healthy cells which were negative for both PI and annexin V-FITC. Counts in Q4 were early apoptotic cells that were only positive for Annexin V-FITC, while counts in Q2 were late apoptotic or dead cells which are positive for both PI and annexin V-FITC. (**E**) Shown are statistical analyses of three independent experiments determining apoptotic populations within CRC cells displaying altered expression of NOV. **p < 0.01. Error bars are standard deviations.

RKO^NOVkd^ cells exhibited an increased proliferation compared with the control cells, while an inhibitory effect on proliferation was seen in the HT115^NOVexp^ cells (Figure 3A). To analyse the molecular mechanisms underlying the effects on cellular functions in RKO NOV knockdown and HT115 NOV overexpression cells we used RT-PCR to detect the transcript level of cyclin: D1, D3, E, A and B1; cyclin-dependent kinase (CDK): 1, 2, 4 and 6; CDK inhibitor (CKI): P15, P16, P21 and P27; G1/S checkpoint proteins: 53 and Rb; apoptosis signalling pathways regulators: caspase 3, 8, 9, Bcl-2, Fas and Survivin (data not shown). There was no differential expression seen within these genes, except in caspase-3, -8 and -9. Caspase-3 and -8 were remarkably down-regulated as a result of NOV knockdown in RKO cells, while an up-regulation was seen in the HT115^NOVexp^ cells (Figure 3B). Corresponding changes were seen in protein levels of the caspases in the RKO cells but not the HT115 cells (Figure 3C). We then examined the effect of altered NOV expression on the apoptosis and survival of CRC cells using a flow cytometric assay (Figure 3D and 3E). There was no obvious effect on apoptosis seen in the CRC cells under general culture condition, i.e. DMEM supplemented with 10% foetal bovine serum (Figure 3D1 and 3D2). However, a better survival was seen in RKO^NOVkd^ cells when they were deprived of the serum. A decreased proportion of apoptotic cells (including both early and late apoptotic cells) were seen in RKO NOV knockdown cells (29.04%) compared to the control cells (48.56%). In contrast, an increased proportion of apoptotic cells were seen in HT115^NOVexp^ cells (43.44%) compared to the HT115^pEF^ cells (31.36%) (Figure 3D3 and 3D4).

### NOV expression and chemoresistance of CRC

**Figure 4:**
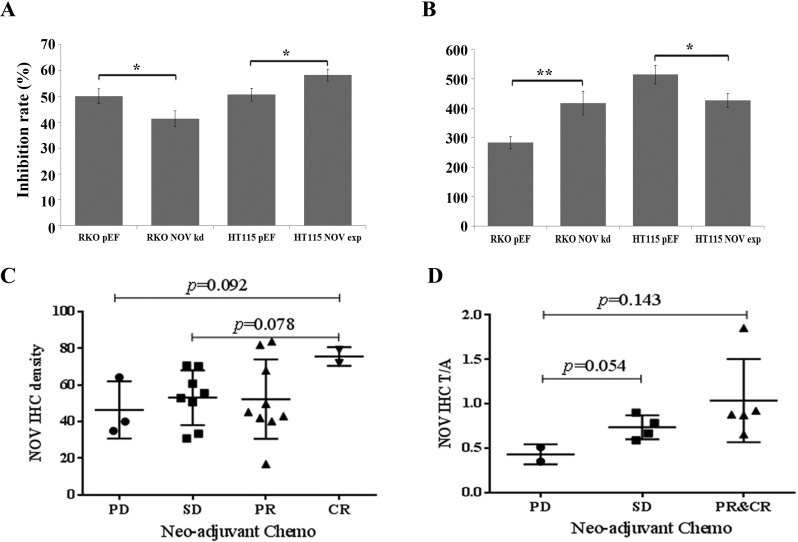
Reduced NOV expression and chemotherapy resistance of CRC cells (**A**) Knockdown of NOV resulted in an enhanced resistance to the treatment of 5-FU, while the same 5-FU treatment achieved a greater inhibition rate in the HT115^NOVexp^ cells compared to their respective controls. The inhibition rates were calculated for the cells treated with 5-FU against their corresponding untreated control cells. (**B**) Proliferative rates in untreated controls. Shown are representative results from three independent experiments. Error bars represent standard error of mean. * represents *p* < 0.05; ** represents *p* < 0.01. (**C**) NOV expression in CRC tumours and the possible link with chemotherapy resistance. IHC was performed for NOV protein in FFPE specimens from diagnostic biopsies. PD = progressive disease; SD = stable disease; PR = partial regression; CR = complete regression. Shown is the intensity of NOV IHC staining, determined using ImageJ and background subtracted. (**D**) Normalized intensity of NOV IHC staining and chemotherapy resistance in CRC. NOV staining in tumours was normalised against their paired adjacent normal colon tissues.

Further to the effect on proliferation and survival of CRC cells, we investigated the influence of altered NOV expression on chemotherapy resistance. 5-FU alone or in combination with other drugs, such as leucovorin calcium, oxaliplatin, irinotecan, bevacizumab and cetuximab, is considered as first-line or second-line chemotherapy regimen in CRC [19]. CRC cells were treated with 5-FU (100 μg/ml) for three days. RKO NOV knockdown cells exhibited a reduced response to the treatment of 5-FU, while NOV overexpression in HT115 cells enhanced the inhibition of cell growth (Figure 4A and 4B).

We then examined the expression of NOV in CRC tumour samples from biopsies of patients who received Neo-adjuvant chemotherapy, using immunochemical staining. There were 22 tumour samples and 11 paired adjacent normal colon tissues. According to the post-operative clinic-pathological assessment, three patients had a progressive disease (PD) after the neo-adjuvant chemotherapy; eight patients had stable disease (SD); nine patients had partial response (PR) and two patients had complete response (CR). Higher staining of NOV was seen in the tumours with CR to the chemotherapy, *p* = 0.078 vs. the SD tumours and *p* = 0.092 vs. the PD tumours (Figure 4C). Following a normalisation against NOV staining in the corresponding adjacent normal colon tissues, a stepwise increase was seen for the NOV staining in tumours with SD (*p* = 0.054), PR and CR tumours (*p* = 0.143) compared with its staining in the progressive tumours (Figure 4D).

### JNK pathway in NOV-regulated cell proliferation, survival and invasion of CRC cells

**Figure 5:**
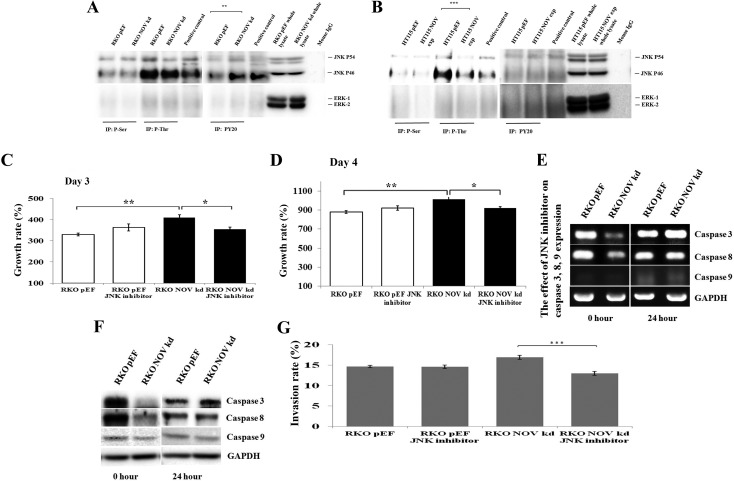
JNK pathway in the NOV-regulated proliferation, survival and invasion of CRC cell lines Activation of JNK and ERK was determined from the phosphorylation of their tyrosine, serine and threonine residues by immunoprecipitation and Western blot (**A**, RKO and **B**, HT115). Positive controls consisted of RKO and HT115 wild type cells treated with 10 mM Sodium orthovanadate and 0.8% (vol/vol) hydrogen peroxide for 10 minutes. JNK inhibitor diminished the pro-proliferative effect of NOV knockdown in RKO cells over a period of three days (**C**) and four days (**D**). The NOV-regulated expression of Caspase -3 and -8 was also blocked by the addition of JNK inhibitor (**E**, RT-PCR and **F**, Western blot). (**G**) NOV knockdown promoted invasion in RKO cells was suppressed by the JNK inhibitor. * represents *p* < 0.05, ** represents *p* < 0.01 and *** represents *p* < 0.001. Error bars represent standard error of mean.

Recent studies have shown the JNK pathway is involved in NOV-regulated or coordinated cellular functions. For example, JNK pathway has been shown to mediate NOV-induced CCL2 expression in rat brain astrocytes [20]. NOV enhances bone morphogenetic protein-4 expression and bone nodule formation in osteoblasts, via integrin-linked kinase, p38, JNK, and AP-1 signalling pathways [21]. Following an initial assessment of signal transduction through MAPK pathways using a Kinex^TM^ antibody microarray (Supplementary Figure 4), changes were observed in phosphorylation of ERK1/2 and JNK, but not p38, in the RKO^NOVkd^ and HT115^NOVexp^ cells. To determine the possible signalling pathways mediating the effect of NOV, immunoprecipitation and Western blot were used to assess the effect of NOV on the threonine, serine and tyrosine phosphorylation of JNK and ERK in RKO and HT115 cells. The results indicated that tyrosine phosphorylation of JNK was increased in RKO NOV knockdown cells compared with control cells (*P* < 0.01) (Figure 5A), while threonine phosphorylation of JNK decreased in HT115^NOVexp^ cells (*P* < 0.001) (Figure 5B). No difference was observed in the phosphorylation of threonine, serine and tyrosine residues of ERK in both RKO and HT115 cells (Figure 5A and 5B). The addition of JNK inhibitor Anthra (1 μM) blocked NOV knockdown-promoted cell proliferation (Figure 5C). We then determined the expression of Caspase -3 and -8 in the RKO^NOVkd^ cells which were exposed to the Anthra for 24 hours. The resultant down-regulation of Caspase -3 and -8 in RKO^NOVkd^ cells diminished with the use of the treatment of Anthra (Figure 5E and 5F). Furthermore, NOV knockdown-promoted invasiveness of RKO cells was also suppressed by the JNK inhibitor (Figure 5G).

## DISCUSSION

Our present study suggests that NOV is a putative tumour suppressor for CRC, as higher NOV expression was detected in the epithelial cells within normal colon tissue compared to CRC tumours. Furthermore, a higher expression of NOV was also observed in the colon tissues compared with rectum. A down regulation of NOV expression is evident in chronic myeloid leukaemia, glioma, malignant adrenocortical tumours and melanoma [18, 22–25], whilst NOV has been found to be up regulated in human prostate cancer, chondrosarcomas, Wilms’ tumour and cervical cancer [16, 26–28]. Our study also linked increased local invasion of CRC tumours (T3 and 4) with elevated levels of NOV transcript levels, though the IHC did not corroborate this. Another recent study assessed the expression of NOV in a cohort of 126 CRC tumours, showing that higher expression in some CRC tumours was associated with poorer survival as well as local invasion [17]. In our current study however, a reduced expression of NOV was seen in CRC tumours compared with paired adjacent normal colon tissues. This is in line with findings from analyses of other GEO data (Supplementary Figure 1). Our current finding was also supported by immunochemical staining of NOV protein in CRC tumours together with paired adjacent normal colon tissues. Certainly, micro-dissected cancerous cells from CRC frozen sections and normal mucosa from normal colonic tissue (either paired or non-paired) and subsequent quantification of gene expression using real time PCR, gene expression micro-array or RNA sequencing will be helpful to settle this controversy. Overexpression of NOV has been shown in metastatic melanoma cells when compared with its expression in primary melanoma. The overexpression of NOV enhanced the adhesion of melanoma cells to laminin and vitronectin through an up regulation of integrin α7β1 and αVβ5 [29]. This contrasts to its reduced expression in primary melanoma, which is associated with promoted invasiveness of melanoma cells at primary sites [18]. This suggests that NOV is a putative tumour suppressor for CRC, and together with previous reported roles in different malignancies, that a differential role can be played by NOV in different organs and tissues. These contradictory findings in different malignancies also suggest that NOV plays diverse and dynamic roles during disease progression, which can be tissue specific or disease specific.

CCNs are matricellular proteins and can be secreted into the extracellular matrix. Our immunochemical staining showed that NOV protein was mostly confined in the cytoplasm of epithelial cells and was reduced in the CRC cancer cells. NOV has been detected in culture medium, extracellular matrix (ECM) and at the cell membrane [30–32], whilst CCN1 has also been detected in culture medium [33]. Our previous study has shown confined staining of CCN4, CCN5 and CCN6 at the cell membrane in normal colorectal epithelial cells, while an increased staining of CCN4 and CCN6 was seen in CRC tumours [12]. A localization of CCN5 in nuclei was also observed in rat and human tissues [34].

To expand our understanding of the role played by NOV in CRC, we examined the influence of NOV on cellular functions of CRC cell lines. The altered expression of NOV resulted in contrasting effects on their proliferation and invasion, but elicited little effect on adhesion and migration. Corresponding changes were also seen in the activated MMP-2/-9 in the NOV knockdown and overexpression cells which were consistent with their contrasting impacts on the invasiveness of the two CRC cell lines. These results indicate that NOV mediates an inhibitory effect on the proliferation and invasion of CRC cells which is in line with the reduced expression observed in the CRC tumours and the corresponding implication for distant metastases. However, Udea *et al* showed that NOV was able to enhance invasiveness of other CRC cell lines (COLO205 and SW480) [17]. It suggested that a different molecular machinery underlying NOV related functions may be utilised in certain CRC tumour cells which is yet to be investigated. For instance, differential expression profile of NOV interacting molecules may divert the response in different cell lines or cancers.

In addition to the effect on cell proliferation, the altered expression of NOV did influence the survival of CRC cells under stress. In the present study, the CRC cells were cultured in a medium with a deprivation of serum which generally induced apoptosis after a culture period of 48 hours. The loss of NOV resulted in a better survival in the RKO cells while more apoptotic cells were seen in the HT115 NOV overexpression cells. These observations supported by a reduced expression of caspase-3 and -8 prompted further investigation of a possible link between reduced expression of NOV and chemoresistance.

Fluorouracil (5-FU)-based chemotherapy is generally considered as the first-line treatment for advanced CRC [35]. Response rates to 5-FU treatment are between 10–20% in the advanced CRC [36], and a better response rate has been reported using combined chemotherapy regimens of 5-FU and oxaliplatin which is 40–50% [37]. In the current study, we examined the responses to 5-FU in CRC cell lines with altered expression of NOV. A reduced effect of 5-FU was seen in RKO NOV knockdown cells while a contrasting change was observed in HT115 NOV overexpression cells.

A further examination of NOV expression in a cohort of biopsy samples from patients who received neo-adjuvant chemotherapy, we observed that CRC tumours with reduced NOV expression tended to be less responsive, though not significantly so, to neo-adjuvant chemotherapy. Interestingly, a ratio of NOV staining in CRC tumours against their paired adjacent normal colon tissues showed a stepwise increase when the tumours were more responsive to the chemotherapy. Its predictive value should be further investigated in a large cohort of CRC patients who received chemotherapy. Quantitative analysis of NOV transcripts or protein in micro-dissected tumour together with paired adjacent normal colon tissues will provide more precise confirmation of the findings in the present study.

NOV has been shown to inhibit Wnt/β-catenin signalling pathway through the suppression of bone morphogenetic protein-2 (BMP-2) activity [38]. In certain myogenic cells the NOV-Notch association exhibits a positive effect on the Notch signalling pathway and suppresses cellular differentiation [39]. Recent studies have shown that JNK is also involved in NOV mediated effects on cellular functions. For example, NOV promoted CCL2 production by Rho/ROCK/JNK/NF-κB pathway in rat brain astrocytes, and played a role in neuro-inflammation [20]. NOV was also able to promote bone formation by up-regulating BMP-4 expression in osteoblasts through integrin-linked kinase, p38, JNK, and AP-1 signalling pathways [21]. To assess the involvement of JNK in the NOV-induced effect on CRC, we determined the activation of JNK in the NOV knockdown and overexpression cells. Tyrosine phosphorylation of JNK was increased in RKO NOV knockdown cells, while a reduced threonine phosphorylation of JNK was seen in HT115 NOV overexpression cells. More interestingly, an addition of small inhibitor targeting JNK eliminated the effect of NOV knockdown on the proliferation and invasion with corresponding changes seen in the expression of caspase-3/-8 and MMP-2/-9. Furthermore, the JNK inhibitor also prevented chemoresistance in the RKO NOV knockdown cells. It suggests that tyrosine phosphorylated JNK and NOV have joint potential in the prediction of chemoresistance. Targeting these two molecules could be warranted, to provide personalised disease management for patients with CRC.

In summary, NOV is down-regulated in CRC tumours which is associated with disease progression. NOV inhibits the proliferation and invasion of CRC cells by regulating caspase-3/-8 and MMP-2/-9 through JNK pathway. NOV together with JNK are promising predictive markers and therapeutic targets for chemoresistance and personalized disease management of CRC.

## MATERIALS AND METHODS

### Cell lines and culture conditions

Human colon cancer cell lines RKO and HT115 were purchased from the American Type Culture Collection (ATCC, Manassas, VA) and incubated at 37°C, 5% CO_2_ and 95% humidity. Wild-type cells were maintained in Dulbecco's modified Eagle's medium supplemented with 10% fetal calf serum (PAA Laboratories Ltd., Somerset, UK), amphotericin B, penicillin and streptomycin (Sigma-Aldrich Inc., Poole, Dorset, UK). Transfected cells were maintained in regular medium containing 0.5 μg/ml blasticidin. JNK inhibitor, Anthra, was purchased from Tocris Bioscience (Bristol, UK).

### Human colorectal cancer tissues

Human CRC tissues including tumours (*n* = 359) and adjacent background tissues (*n* = 174) were collected immediately after surgery and stored at −80°C. Total RNA extraction was performed for this cohort of frozen tissues. All protocols were approved by the Beijing Friendship Hospital Research Ethical Committee. Written consents were obtained from the patients.

### RNA isolation, reverse transcription-polymerase chain reaction (RT-PCR) and quantitative real time PCR (QPCR)

Total RNA was extracted from confluent cells in a 25 cm^2^ flask using a RNA isolation (TRI) reagent following the standard protocol (Sigma-Aldrich, Dorset, UK). Fresh frozen tissues were also first homogenized in TRI reagent using a hand held homogenizer. First strand cDNA was synthesized from 1 μg RNA using a reverse transcription kit (BioRad, UK). NOV mRNA expression in CRC tissues was determined using real time PCR and an Ampliflor™-based PCR method, in which a 6-carboxy-fluorescine-tagged Uniprimer™ (Biosearch Technologies, Inc.) was used as a probe together with a pair of target specific primers. The reverse primer contained a Z-sequence (actgaacctgaccgtaca) which is complimentary to the unique sequence in the Uniprimer. Glyceraldehyde 3-phosphate dehydrogenase (GAPDH) was used as a housekeeping gene. Primers used in the present study are listed in Table 2.

**Table 2:** Primer sequences

Gene	Forward primers (5′-3′)	Reverse primer (5′-3′)
*GAPDH*	GGCTGCTTTTAACTCTGGTA	GACTGTGGTCATGAGTCCTT
*GAPDH (Q-PCR)*	CTGAGTACGTCGTGGAGTC	ACTGAACCTGACCGTACACAGAGATGACCCTTTTG
*NOV*	CTCCAAGAAAAGTTGAGGTG	CTGGCTTCTTGACTATTTGC
*NOV (Q-PCR)*	CTGTGAACAAGAGCCAGAG	ACTGAACCTGACCGTACACTTGAACTGCAGGTGGAT
*NOV ribozyme*	CTGCAGCGCTGAGTCGCAGCGACCTGTCCCAGGACTGATGAGTCCGTGAGGA	ACTAGTGCCTTTGCCTGACCTTCCTGCTTCTCCATTTCGTCCTCACGGACT
*NOV CDs*	ATGCAGAGTGTGCAGAGCA	CATTTTCCCTCTGGTAGTCTTCA
*Caspase3*	GGCGTGTCATAAAATACCAG	ACTGAACCTGACCGTACAACAAAGCGACTGGATGAA
*Caspase8*	AAGCCCAAGCTCTTTTTC	ACTGAACCTGACCGTACAGTTACTGCCAGGGGACTC
*Caspase9*	GGCTGCTTTTAACTCTGGTA	GACTGTGGTCATGAGTCCTT

### Immunochemical staining of NOV in CRC Formalin Fixed Paraffin Embeded (FFPE) specimens

FFPE sections including 45 CRC tumours and 27 adjacent normal colorectal tissues were provided by the Beijing Friendship Hospital. FFPE specimens from biopsies in a group of patients who received neo-adjuvant chemotherapy at the Beijing Friendship Hospital were also used for assessing the expression of NOV by immunochemical staining. All patients in this group had liver metastases at the diagnosis. The tissue samples were from biopsies before the neo-adjuvant chemotherapy. In total, there were 22 tumour samples with 11 tumours having paired adjacent normal colon tissues in this group of specimens. Response to neo-adjuvant chemotherapy was evaluated using RECIST criteria version 1.1 [40]. All protocols were approved by the Beijing Friendship Hospital Research Ethical Committee. Written consents were obtained from the patients. A verification and HE staining were undertaken by pathologists at the Beijing Friendship Hospital. In brief, IHC staining of NOV was performed for these FFPE specimens following a standard procedure. Following a de-waxing and rehydration in PBS, antigens were retrieved using 1mM EDTA (pH 8.0) in a microwave for 20 minutes. After cooling in tap water and washing in PBS, the sections were then blocked in 5% horse serum for 2 hours, before an overnight incubation at 4°C with anti-NOV antibody (0.5 μg/ml, ab137677, Abcam). A secondary biotinylated antibody and the avidin biotin complex was used to detect NOV expression in accordance with the Vectastain Universal Elite ABC kit protocol (Vector Laboratories, Peterborough, UK), and the end product was developed with 3,3′-diaminobenzidine (DAB). The sections were then counterstained with Gill's hematoxylin. The intensity of staining was independently assessed by the authors using ImageJ (http://imagej.nih.gov/ij/).

### Construction of ribozyme transgene targeting human NOV and the establishment of corresponding stable transfectants

Anti-human NOV hammerhead ribozymes were designed and generated using Zuker RNA mFold program [41]. The ribozymes were produced and cloned into the pEF6/V5-His TOPO vector (Invitrogen, Paisley, UK). The full-length coding sequence of human NOV was amplified from a human placenta cDNA library. The NOV coding sequence (CDS) was also cloned into the pEF6/V5-His TOPO vector. Constructed ribozyme transgenes, NOV expression vector and empty plasmid vector were transfected into RKO and HT115 cells, respectively, using an Easyject Plus electroporator (EquiBio, Kent, UK). After one week of selection in DMEM containing 5 μg/ml blasticidin followed by another week of culture in DMEM containing a maintenance concentration of 0.5 μg/ml blasticidin, the transfected cells were verified for the expression of NOV and were subsequently used for the following experiments.

### Western blot analysis

Following lysis of cells, the protein concentration was determined using the DC protein assay kit (Bio-Rad) and the ELx800 spectrophotometer (Biosearch Technologies, Inc.). Equal amount of each protein samples were separated using SDS-PAGE followed by electro blotting onto a nitrocellulose membrane. The proteins were then probed with the anti-NOV antibody (1:2,000, Abcam Ltd.) and anti-GAPDH antibody (1:2,000, Santa Cruz Biotechnology, Inc.), as a house keeping gene control, followed by a peroxidase-conjugated secondary antibody (1:2,000, Sigma). Protein bands were visualized using a chemiluminescence detection kit (Luminata, Millipore) and photographed using a Syngene imager (Syngene International Ltd.).

### Kinex^TM^ antibody microarray

To prepare cell lysates for use with the Kinex^TM^ antibody microarray (Kinexus Bioinformatics Corporation, Vancouver, Canada), two T75 flasks of cells were grown to approximately 90% confluence. The cells were washed twice with PBS before adding 20 ml of DMEM which was supplemented with 2% FCS. Following a 2-hour incubation at 37°C, the cells were lysed in a lysis buffer containing 100mM tris, 10% 2-mercaptoethanol, 1% NP-40, 50 mM NaFI, 2 mM 4-(2-aminoethyl) benzenesulfonyl fluoride (AEBSF), 14 μM E-64, 130 μM bestatin, 1 μM leupeptin, 0.3 μM aprotinin, and 1mM EDTA.

### *In vitro* cell growth assay

The IC50 of 5-Fluorouracil (5-FU) was determined using a colorimetric method to quantify cells [42]. In brief, 3,000 cells were seeded into two 96-well plates in culture medium and with different concentrations of 5-FU. Cells were incubated for 1 and 3 days before being fixed with 4% formaldehyde, and stained with 0.5% crystal violet. The stain was then dissolved in 10% acetic acid prior to the colorimetric detection of cell density using the ELx800 spectrophotometer at a wavelength of 540 nm.

### *In vitro* invasion assay

Invasion assays were performed following a previously described procedure [43]. Transwell inserts with 8 μm pores were pre-coated with 50 μg of Matrigel Matrix Basement Membrane (BD Bioscience, Oxford, UK) and air-dried. Following rehydration, 30,000 cells were seeded to each insert and also control wells on a 24-well plate. After an incubation of 72 hours, cells that had migrated through the matrix to the other side of the insert were fixed, stained, counted and then dissolved with 10% acetic acid. Cell density was determined by reading the absorbance at 540 nm with the ELx800 spectrophotometer.

### Cell-matrix adhesion assay

The cell-matrix adhesion assay was conducted as previously described [43]. A 96-well culture plate was pre-coated with 5 μg of Matrigel and air-dried. The Matrigel was rehydrated before seeding 30,000 cells to each well. Non adherent cells were washed off using a PBS buffer following an incubation of 40 minutes. Adherent cells were then fixed and stained. The number of adherent cells were counted before the absorbance of the crystal violet stain was determined by dissolving in 10% acetic acid and results were read on a ELx800 spectrophotometer.

### Wound healing assay

The assay was performed as previously described [43]. Cells were seeded onto 24-well plates and incubated overnight. The monolayer of cells was scraped with a 10μl pipette tip. The migration of cells was monitored and photographed using the EVOS system, a fluorescent inverted microscope system equipped with an on-stage incubator (Life Technologies Ltd, Paisley, UK), and the migration of cells was determined using ImageJ.

### Immunoprecipitation (IP) and western bolt analysis

The cells were lysed and the protein concentration was determined following the aforementioned procedure. Equal amounts of protein samples (160μg/80μl) were incubated with the anti-phosphotyrosine antibody (PY20, 1:500, Santa Cruz Biotechnology, Inc.), anti-phosphoserine antibody (p-Ser, 1:500, Santa Cruz Biotechnology, Inc.), and anti-phosphothreonine antibody (p-Thr, 1:500, Santa Cruz Biotechnology, Inc.) at 4°C for 1 hour respectively, followed by the addition of 15μl protein A/G plus-agarose beads (Santa Cruz Biotechnology, Inc.) and an incubation of 1 hour. The samples were washed with SDS-free lysis buffer twice before they were boiled with 1× sample buffer (Sigma). Proteins were probed with the anti-JNK antibody (1:500, Santa Cruz Biotechnology, Inc.), and anti-ERK antibody (1:500, Santa Cruz Biotechnology, Inc.), followed by a peroxidase-conjugated secondary antibody (1:2,000, Sigma). Protein bands were visualized using a chemiluminescence detection kit (Luminata, Millipore) and photographed using a Syngene imager (Syngene International Ltd.).

### Gelatin zymography assay

To prepare conditioned medium from cancer cells, 2 × 10^6^ cells were seeded into a T25 flask. Following overnight culture, the cells were washed once with 1 × PBS followed by another wash with serum-free DMEM, and were then incubated in serum-free DMEM medium for 6 hours. The conditioned medium was collected. Samples were prepared in non-reducing sample buffer (0.625 mM Tris-HCl, 10% glycerol, 2% SDS, and 2% bromophenol blue), and were then separated using SDS-PAGE containing 0.25% gelatin (Sigma-Aldrich Inc, USA). The gels were re-natured twice using washing buffer (2.5% triton X-100 and 0.02% NaN_3_) and then incubated for 48 hours at 37°C in an incubation buffer (50 mM Tris-HCl, 5 mM CaCl_2_, and 0.02%NaN_3_). The gels were stained with coomassie blue for 30 minutes and de-stained twice with de-stain buffer (10% acetic acid and 25% ethanol). The gels were then photographed using a Syngene imager (Syngene International Ltd.).

### Flow cytometric analysis of apoptosis

For apoptosis analysis, 8×10^5^ cells were seeded into T25 flasks and cultured in DMEM supplemented with 10% FCS, while 2 × 10^6^ cells were seeded into T25 flasks which were cultured in serum free DMEM. Following 48-hour culture, both adherent and cells present in the medium were harvested. The apoptotic population was determined using the Annexin V kit (Santa Cruz Biotechnology) and analyzed using PartecCyFlow^®^ SL flow cytometry and the accompanying FloMax software package (Partec GmbH, Münster, Germany) [44].

### Statistical analysis

Statistical analyses were performed using SPSS (IBM Corporation, Armonk, NY). *T*-test and Mann Whitney tests were used for normally distributed data and non-parametric data respectively. A *p*-value of < 0.05 was considered statistically significant.

## SUPPLEMENTARY MATERIALS FIGURES AND TABLES


