# Supplementary material for: Reduced NOV expression correlates with disease progression in colorectal cancer and is associated with survival, invasion and chemoresistance of cancer cells
Source: Oncotarget. 2017 Feb 17;8(16):26231–44. doi: 10.18632/oncotarget.15439 (PMC5432252; doi:10.18632/oncotarget.15439)
Supplement: Supplementary file 1 [file oncotarget-08-26231-s001.pdf]

Reduced NOV expression correlates with disease progression in colorectal cancer and is associated with survival, invasion and chemoresistance of cancer cells

Supplementary Materials

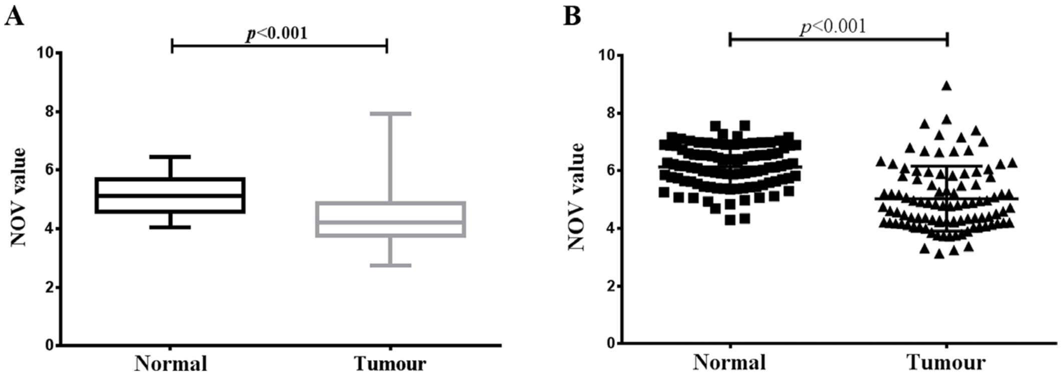

Supplementary Figure 1: NOV expression in human CRC was analysed in two gene expression array datasets. (**A**) NOV expression in CRC tumours (*n* = 566) was lower compared with its expression in normal colon tissues (*n* = 19) [45]. (**B**) NOV expression was reduced in CRC tumours compared with paired adjacent normal colon tissues (*n* = 98) [46]. Error bars are standard deviations.

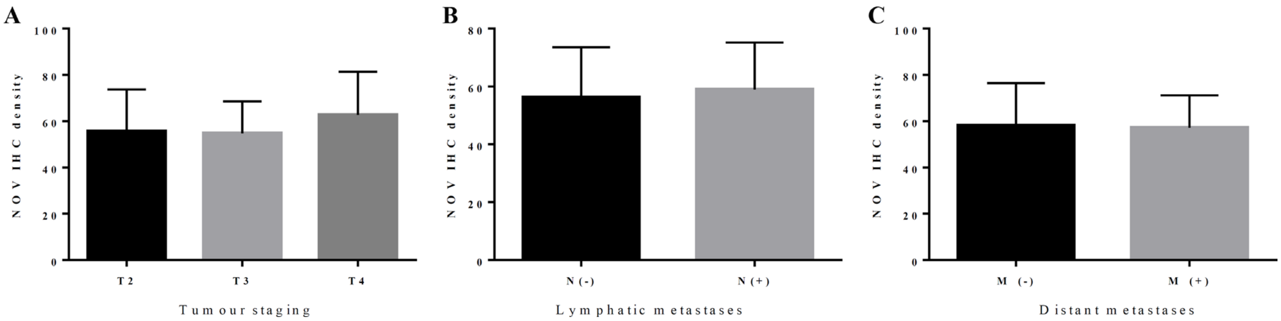

Supplementary Figure 2: Semi-quantitative analysis of NOV IHC density in CRC paraffin sections. (**A**) NOV expression in CRC tumours of different tumour stages. (**B**) NOV expression in primary tumours with lymph node metastases. (**C**) NOV IHC staining in primary tumours with distant metastases. Error bars are standard deviations.

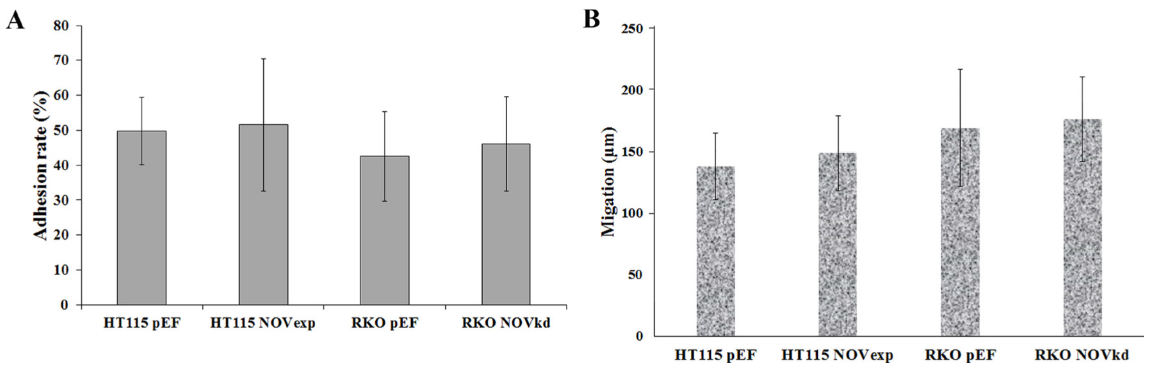

Supplementary Figure 3: Influence of altered NOV expression on adhesion and migration of CRC cell lines. (**A**) Adhesion of HT115 and RKO cells with overexpression and knockdown of NOV. Six repeats were included for each cell lines in each experiment. (**B**) Average migrated distance of NOV overexpression and RKO knockdown cells over a period of six hours. Three repeats were included for each cell line in the migration assay. Shown are representative results from three independent experiments. Error bars are standard deviations.

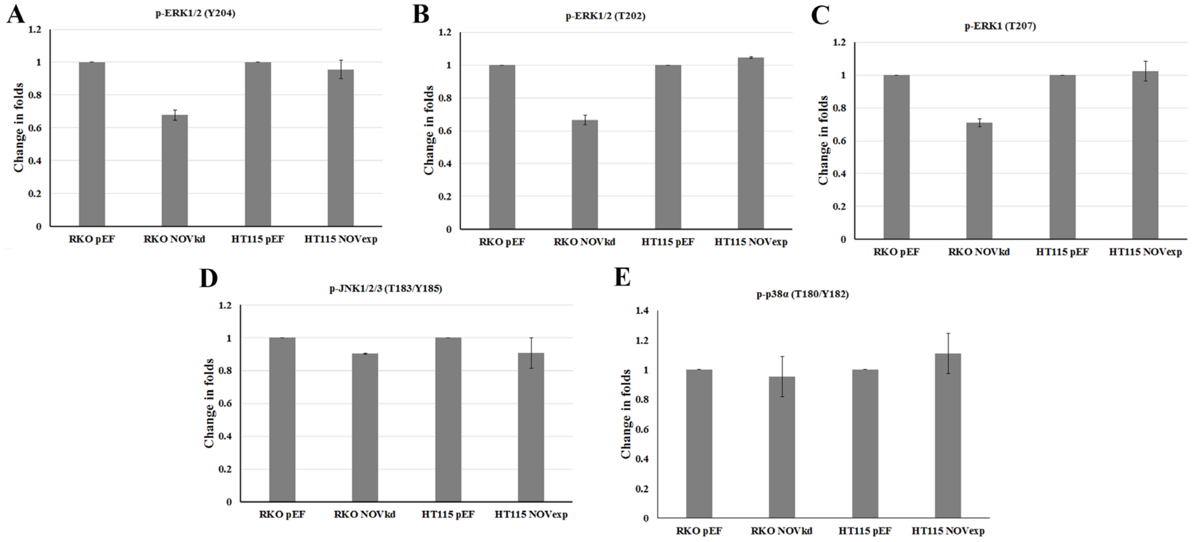

Supplementary Figure 4: Effect on signal transduction through ERK, JNK and p38 pathways was determined using a Kinex^{TM} antibody microarray. Shown are change in intensity of phosphorylation staining of ERK1/2 at Y204 (**A**), T202 (B) and T207 (**C**); T183/Y185 of JNK (**D**) and T180/Y182 of p38 in RKO and HT115 control and transfected cells. Antibodies targeting the same phosphorylated protein were pooled and changes (in fold) were calculated against the corresponding controls. Error bars are standard deviations.

Supplementary Table 1: Summative information of Immunochemical staining of NOV in CRC tissues

		n	IHC intensity (mean ± SD)
Tumor		45	60.7 ± 19.1
Adjacent normal		27	77.6 ± 24.2
T stage	T2	13	55.6 ± 18.1
	T3	15	54.8 ± 13.8
	T4	12	62.7 ± 18.7
	Missing	5	
N stage	N (−)	22	56.2 ± 17.4
	N (+)	18	59.0 ± 16.3
	Missing	4	
M stage	M (−)	26	58.1 ± 18.2
	M (+)	13	57.2 ± 14.0
	Missing	6	
